# Determination of the composition of heterogeneous binder solutions by surface plasmon resonance biosensing

**DOI:** 10.1038/s41598-021-83268-z

**Published:** 2021-02-11

**Authors:** Jimmy Gaudreault, Benoît Liberelle, Yves Durocher, Olivier Henry, Gregory De Crescenzo

**Affiliations:** 1 Department of Chemical Engineering, Polytechnique Montréal, Centre-Ville Station, P.O. Box 6079, Montreal, QC H3C 3A7 Canada; 2 Life Sciences | NRC Human Health Therapeutics Portfolio, Building Montreal-Royalmount, National Research Council Canada, Montreal, QC H4P 2R2 Canada

**Keywords:** Biochemistry, Biophysical chemistry, Pharmaceutics, Assay systems

## Abstract

Surface plasmon resonance-based biosensors have been extensively applied to the characterization of the binding kinetics between purified (bio)molecules, thanks to robust data analysis techniques. However, data analysis for solutions containing multiple interactants is still at its infancy. We here present two algorithms for (1) the reliable and accurate determination of the kinetic parameters of N interactants present at different ratios in N mixtures and (2) the estimation of the ratios of each interactant in a given mixture, assuming that their kinetic parameters are known. Both algorithms assume that the interactants compete to bind to an immobilized ligand in a 1:1 fashion and necessitate prior knowledge of the total concentration of all interactants combined. The effectiveness of these two algorithms was experimentally validated with a model system corresponding to mixtures of four small molecular weight drugs binding to an immobilized protein. This approach enables the in-depth characterization of mixtures using SPR, which may be of considerable interest for many drug discovery or development applications, notably for protein glycovariant analysis.

## Introduction

In the last three decades, Surface Plasmon Resonance (SPR)-based biosensors have become the method of choice to study binding between an immobilized (bio)molecule—referred to as the ligand—and its soluble partner—the analyte. The said analyte may be purified^1^ or in a crude extract^2–4^. Through the years, important advances were made in the development of optimized experimental protocols^5^ and data analysis methods^6–12^. Robust data analysis software packages were created and contributed to the success of this label-free technology^13–16^. Recent efforts have brought the SPR technology at the forefront of drug development for (i) the off-line^17^ or at-line^18^ characterization of the critical quality attributes of biotherapeutics or (ii) for the screening of large libraries of drug candidates^19^. On that specific note, our group recently demonstrated that the throughput of a SPR-based experiment could be improved by injecting two analytes simultaneously, since the binding kinetics of both analytes could be identified in only one set of experiments^10,12^. These methods have yet to be expanded to the study of more than two analytes. We also demonstrated that analyte concentration could be identified from a SPR sensorgram, provided that the kinetics of its interaction to its immobilized ligand are known^20^. However, this approach, which uses parameter identification techniques, only considered pure analyte injections.

The SPR identification of the kinetic parameters for each individual analyte within a complex analyte mixture would also be of great interest to determine the individual kinetic parameters of various (bio)molecules that are hard to separate from each other. Such is the case, for instance, for the various glycoforms of therapeutic monoclonal antibodies produced in mammalian cell cultures, for which binding to their receptors is affected by their glycosylation state^21–27^. Hence, SPR data analysis techniques that can extract specific kinetic parameters for different glycoforms are required for the analysis of antibody SPR sensorgrams.

The present study aims at providing a twofold framework enabling the treatment of mixtures of more than two analytes. In a first part, we present an algorithm to extract meaningful kinetic parameters from SPR sensorgrams corresponding to the injections of $N$ mixtures of $N$ analytes. In a second part, we exploit the prior knowledge of the kinetic parameters of a set of analytes to estimate the composition of a mixture of these analytes by performing a sensorgram analysis. We validated our algorithms using a well-characterized model system^10–12,20,28^, which consists of four small molecular weight drugs all binding to immobilized carbonic anhydrase II (CAII) with different kinetics and affinities.

## Materials and methods

### Materials

Biacore T100 biosensor, research-grade CM5 sensor chips, HBS-EP buffer (HEPES Buffered Saline with 30 mM EDTA [ethylenediaminetetraacetic acid] and 0.5% (v/v) surfactant P20), 70% v/v glycerol in water and ethanolamine were purchased from Cytiva (formerly GE Healthcare, Baie d’Urfe, QC). N-ethyl-N′-(3-dimethylaminopropyl) carbodiimide (EDC), N-hydroxysuccinimide (NHS), carbonic anhydrase isozyme II (CAII) purified from bovine erythrocytes, glacial acetic acid, sodium acetate, 4-carboxybenzenesulfonamide (CBS), 1,3-benzene-disulfonamide (BDS), sulfanilamide, furosemide and dimethyl sulfoxide (DMSO) were purchased from Sigma-Aldrich Canada Ltd (Oakville, ON).

### Biosensor surface preparation

The biosensor surface preparation was performed according to previously published protocols^28^. Briefly, sensor chip surfaces were activated at 25 °C with an injection of 1:1 0.4 M EDC and 0.1 M NHS during 7 min at 20μL/min. CAII (0.1 g/L in 10 mM acetate buffer, pH 5.0) was then injected (30 s pulses at 20μL/min) until a density of immobilized CAII of approximately 5000RU was reached. Next, a 4 min injection of ethanolamine (1 M, pH 8.5) at 20μL/min was performed to block the remaining activated sites. Ultimately, the whole process led to approximately 4500RU of immobilized CAII. Blank surfaces were generated using the same activation/deactivation protocol but omitting CAII injections. After ligand immobilization and blank surface preparation, the system was extensively primed with running buffer (HBS-EP containing 3% of DMSO) and normalized with a solution of 70% v/v glycerol in water.

### Biacore sample injections

#### Analyte preparation

HBS-EP containing 3% of DMSO (HBS-EP + 3%DMSO) was used as running buffer. All the analytes were dissolved in DMSO and aliquoted at the following concentrations: [CBS] = 596.42 mM, [BDS] = 118.51 mM, [sulfanilamide] = 563.30 mM, [furosemide] = 3075.30 mM. For each experiment, one aliquot of each analyte was dissolved in HBS-EP + 3%DMSO to reach the following concentrations: [CBS] = 52.98 μM, [BDS] = 10.49 μM, [sulfanilamide] = 50.27 μM, [furosemide] = 50.52 μM. Each of these stock solutions were then used to prepare the different dilutions and mixtures used in this study.

#### Single-analyte injections

All injections were performed in duplicate at a flow rate of 85μL/min. The data collection rate was set to 10 Hz and the temperature to 18 °C. For ‘classical’ single-analyte kinetic experiments, CBS, BDS, sulfanilamide and furosemide were injected alone at 5 different concentrations ranging from 1.06 μM to 52.98 μM, 210 nM to 10.49 μM, 1.01 μM to 50.27 μM and 1.01 μM to 50.52 μM, respectively. For double-referencing purposes, 2 buffer injections per analyte were also performed. The injection time was set to 60 s for all analytes and the dissociation time was 420 s for CBS, 240 s for BDS and sulfanilamide and 300 s for furosemide. No regeneration step was needed between injections as the dissociation was complete in every case. This agrees with previous studies that used this system ^10–12,20,28^. The SPR biosensor used in this study (Biacore T100) uses a detection mode based on varying the incident angle while keeping the wavelength constant. The biosensor uses the geometry proposed by Kretschmann and Raether^29,30^.

#### Multiple-analyte injections

The four analytes were combined to create 12 mixtures each containing the 4 analytes, as described in Table 1. Four mixtures were rich in one analyte (70–10–10–10 mixtures, A–D), 4 mixtures had a dominating presence of one analyte, but at lower purity (40–20–20–20 mixtures, E–H) and 4 mixtures were created with random compositions (Random mixtures, I–L). The mixture creation was intended to mimic different types of mixtures one could wish to study in a practical application. For all mixtures, the analyte and the buffer injection phases were set to 240 and 320 s, respectively. Each mixture was injected in duplicate at 18 °C with a flow rate of 85μL/min at 7 different concentrations comprised between 300 nM and 15 μM with a data collection rate of 10 Hz. Again, no regeneration was necessary.

**Table 1:** Molar composition of the 4 single-analyte solutions and 12 mixtures used in this study.

Data set	Mixture	% CBS	% BDS	% Sulfanilamide	% Furosemide
Single-analyte	CBS	100	0	0	0
	BDS	0	100	0	0
	Sulfanilamide	0	0	100	0
	Furosemide	0	0	0	100
70–10–10–10 mixtures	A	70	10	10	10
	B	9	71	10	10
	C	10	10	70	10
	D	10	10	10	70
40–20–20–20 mixtures	E	40	20	20	20
	F	20	40	20	20
	G	20	20	40	20
	H	20	20	20	40
Random mixtures	I	30	32	6	32
	J	43	5	18	33
	K	14	29	26	31
	L	41	18	34	7

For clarity, the mixtures are regrouped in sets named according to the relative fraction of each drug.

### Data analysis

In the case of ‘classical’ single-analyte experiments, each double-referenced set of sensorgrams was fitted to a 1:1 Langmuir model using a standard ‘global fit’ approach. Briefly, sensorgrams corresponding to different concentrations were simultaneously analyzed to obtain a set of global parameter estimates via a least square minimization using a custom program developed with the Matlab R2018b software platform (The Mathworks, Natick, USA)^9^. The parameters obtained with our platform matched closely those obtained with the Biacore T100 Evaluation Software^15^ with deviations ranging from 0 to 2% for CBS, BDS and sulfanilamide and from 1 to 9% for furosemide (data not shown). The algorithms we developed to (1) extract the kinetic parameters from the sets of sensorgrams corresponding to the various analyte mixtures (Tables 1) and (2) estimate the composition of a given mixture based on the prior knowledge of the kinetic constants of each analyte are presented in detail in the next section. Both algorithms assumed that each analyte bound to CAII according to a 1:1 Langmuir model. Fitting the multi-analyte data and estimating mixture composition was also performed using custom-designed Matlab scripts. As sensorgrams corresponding to the different dilutions of the different mixtures needed to be analyzed simultaneously within the fitting procedure, this type of analysis will be further described in the text as the ‘global–global’ approach.

## Multi-analyte modeling and kinetic parameter identification

### The interaction model

We assume a Langmuir 1:1 interaction between each analyte (${A}_{i}$) and the ligand ($L$):

1 $$\begin{array}{c}{A}_{i}+L\begin{array}{c}\stackrel{{k}_{a,i}}{\to }\\ \underset{{k}_{d,i}}{\leftarrow }\end{array}{A}_{i}L, \forall i=1,\dots ,N\end{array}$$

With ${k}_{a,i}$ and ${k}_{d,i}$ corresponding to the association and dissociation rate constants of the ${i}^{th}$ analyte, respectively. For N analytes, the system is described by the following ordinary differential equation (ODE) system:

2 $$\begin{array}{c}\frac{d{R}_{i}}{dt}={k}_{a,i}{F}_{i}{C}_{TOT}{R}_{max,i}\left(1-\sum_{j=1}^{N}\frac{{R}_{j}}{{R}_{max,j}}\right)-{k}_{d,i}{R}_{i},{R}_{i}\left(0\right)=0, \forall i=1,\dots ,N \end{array}$$

$${R}_{TOT}=\sum_{i=1}^{N}{R}_{i}$$

${R}_{max,i}$ is the theoretical SPR response that would be obtained if an infinite concentration of analyte $i$ were injected. ${F}_{i}$ is the fraction of analyte $i$ in the injected mixture and ${C}_{TOT}$ is the total concentration of all analytes. We can then compute the predicted SPR response (${R}_{TOT}$) and the contribution of each analyte to the response (${R}_{i}$) by solving the system of ODE in (2). Furthermore, it can be shown that the contribution of every analyte at equilibrium is given by:

3 $$\begin{array}{c}{R}_{eq,i}=\frac{{R}_{max,i}{C}_{TOT}{F}_{i}{K}_{A,i}}{1+{C}_{TOT}\sum_{j=1}^{N}{F}_{j}{K}_{A,j}} \forall i=1,\dots ,N \end{array}$$

where ${K}_{A,i}$ is the affinity of a given analyte for the immobilized ligand. Note that, for generality purposes, we here consider that each analyte has its own ${R}_{max,i}$.

### Identification of equilibrium parameters

The following algorithm was developed to identify the kinetic parameters (${k}_{a,i}$ and ${k}_{d,i}$) and the corresponding ${R}_{max,i}$ of $N$ analytes. Some conditions on the data set to be analyzed must be met:

- At least $N$ sets of sensorgrams must be recorded with analyte mixtures of different compositions (at least $N$ mixtures).
- The composition of the $N$ mixtures must be known and be linearly independent.
- Each mixture must be injected at least 3 times at different known ${C}_{TOT}$.
- The sensorgrams must reach equilibrium.
- The sensorgrams must return to zero during the dissociation phase.

For a given mixture injected at known dilutions, the observed equilibrium responses can be described analogously to a single-analyte experiment by a set of observed equilibrium parameters (${K}_{A,obs}$ and ${R}_{max,obs}$):

4 $$\begin{array}{c}{R}_{eq,obs}=\frac{{R}_{max,obs}{K}_{A,obs}{C}_{TOT}}{1+{C}_{TOT}{K}_{A,obs}}+{R}_{O}\end{array}$$

When fitting the model, adding an offset parameter (${R}_{O}$) can lead to better fits as it can account for bulk refractive index contributions. ${K}_{A,obs}$ and ${R}_{max,obs}$ can be obtained by simple nonlinear least square regression. This optimization problem and all others in this manuscript were solved using an interior-point algorithm (‘fmincon’ function in MATLAB).

By combining (${R}_{eq,obs}=\sum_{i=1}^{N}{R}_{eq,i}$), we then have:

5 $$\begin{array}{c}{K}_{A,obs}=\sum_{i=1}^{N}{{F}_{i}K}_{A,i}\end{array}$$

6 $$\begin{array}{c}{R}_{max,obs}=\frac{\sum_{i=1}^{N}{F}_{i}{K}_{A,i}{R}_{max,i}}{{K}_{A,obs}}\end{array}$$

Equations (5) and (6) imply that, given the experimental plateau values for the sets of sensorgrams of $N$ mixtures of analytes, one can obtain the individual ${K}_{A,i}$ and ${R}_{max,i}$ by solving two systems of linear equations:

7 $$\begin{array}{c}F{{\varvec{K}}}_{{\varvec{A}}}={{\varvec{K}}}_{{\varvec{A}},{\varvec{o}}{\varvec{b}}{\varvec{s}}}\end{array}$$

8 $$\begin{array}{c}FR={{\varvec{R}}}_{{\varvec{m}}{\varvec{a}}{\varvec{x}},{\varvec{o}}{\varvec{b}}{\varvec{s}}}\cdot {{\varvec{K}}}_{{\varvec{A}},{\varvec{o}}{\varvec{b}}{\varvec{s}}}\end{array}$$

where bold font indicates a matrix or a vector and a dot ($\cdot$) denotes an element-wise multiplication. Each line of ${\varvec{F}}$ corresponds to a mixture and each column to a given analyte (i.e., each line sums to unity). ${{\varvec{K}}}_{{\varvec{A}},{\varvec{o}}{\varvec{b}}{\varvec{s}}}$ and ${{\varvec{R}}}_{{\varvec{m}}{\varvec{a}}{\varvec{x}},{\varvec{o}}{\varvec{b}}{\varvec{s}}}$ are column vectors containing the observed parameters (1 per mixture). Solving (7) for ${{\varvec{K}}}_{{\varvec{A}}}$ returns a column vector containing each ${K}_{A,i}$. Solving (8) for ${\varvec{R}}$, we can obtain the vector of ${R}_{max,i}$ (${{\varvec{R}}}_{{\varvec{m}}{\varvec{a}}{\varvec{x}}}$):

9 $$\begin{array}{c}{{\varvec{R}}}_{{\varvec{m}}{\varvec{a}}{\varvec{x}}}=R\cdot \frac{1}{{{\varvec{K}}}_{{\varvec{A}}}}\end{array}$$

Equations (7) and (8) highlight the need to use mixtures of independent compositions, as the ${\varvec{F}}$ matrix needs to be invertible to solve the systems of linear equations, as previously mentioned.

### Identification of kinetic parameters

During the dissociation phase, only buffer is injected, so that ${C}_{TOT}=0$. If the equilibrium is reached during the analyte injection phase, the system of ODE further simplifies to:

10 $$\begin{array}{c}\frac{d{R}_{i}}{dt}=-{k}_{di}{R}_{i}{, R}_{i}\left(t={t}_{off}\right)={R}_{eq,i}, \forall i=1,\dots ,N\end{array}$$

$${R}_{TOT}=\sum_{i=1}^{N}{R}_{i}$$

Here, ${t}_{off}$ is the time point at which buffer injection starts. This ODE system has a straightforward analytical solution:

11 $$\begin{array}{c}{R}_{i}\left(t\right)={R}_{eq,i} {e}^{-{k}_{d,i}\left(t-{t}_{off}\right)}, \forall i=1,\dots ,N\end{array}$$

$${R}_{TOT}=\sum_{i=1}^{N}{R}_{i}.$$

With the previously obtained estimates of the ${K}_{A,i}$ and ${R}_{max,i}$, we can compute the ${R}_{eq,i}$ for each analyte and each sensorgram in the data set using (3). We then take the integral of the signal during the dissociation phase (11):

$${\int }_{{t}_{off}}^{{t}_{end}}{R}_{TOT}\left(t\right)dt={\int }_{{t}_{off}}^{{t}_{end}}\sum_{i=1}^{N}{R}_{i}\left(t\right)dt={\int }_{{t}_{off}}^{{t}_{end}}\sum_{i=1}^{N}{R}_{eq,i} {e}^{-{k}_{d,i}\left(t-{t}_{off}\right)}dt$$

$$={\left.\sum_{i=1}^{N}\frac{{-R}_{eq,i}}{k_{d,i}} {e}^{-{k}_{d,i}\left(t-{t}_{off}\right)}\right|}_{t={t}_{off}}^{t={t}_{end}}$$

12 $$\begin{array}{c}=\sum_{i=1}^{N}\frac{{R}_{i,eq}}{{k}_{d,i}} \left(1-{e}^{-{k}_{d,i}\left({t}_{end}-{t}_{off}\right)}\right)\end{array}$$

If ${t}_{end}-{t}_{off}$ is sufficiently large, i.e. if the dissociation phase lasts long enough that the observed signal tends to 0 towards the end of the sensorgram, the integral can be simplified to:

13 $$\begin{array}{c}{\int }_{{t}_{off}}^{{t}_{end}}{R}_{TOT}\left(t\right)dt=\sum_{i=1}^{N}\frac{{R}_{eq,i}}{{k}_{d,i}}\end{array}$$

The integral in (13) can be computed numerically using the trapezoidal method, for example. We can obtain an estimate of the dissociation rate constants (${k}_{d,i}$) by a simple linear regression over all sensorgrams of all mixtures of the data set. Note that the ${k}_{d,i}$ can be said to be ‘global–global’ parameters because they apply to all the sensorgrams of all the analyte mixtures in the data set. We can finally obtain an estimate of the association rate constants (${k}_{a,i}$) from the definition of the affinity:

14 $$\begin{array}{c}{k}_{ai}={K}_{Ai} {k}_{di}\end{array}$$

In theory, we then have extracted all the relevant kinetic parameters of the $N$ analytes. However, as $N$ increases, more sensorgrams are required. The parameter identification can be sensitive to the amount of noise contained in the sensorgrams. To make the approach more robust, two nonlinear optimization routines were added.

The first optimization pertains to the dissociation phase. Using the previously estimated ${k}_{d,i}$ as a starting point, we can obtain better estimates by least squares:

15 $$\begin{array}{c}{R}_{i,pred}\left(t\right)={R}_{eq,pred,i} {e}^{-k{d}_{pred,i}\left(t-{t}_{off}\right)}\end{array}$$

$${R}_{TOT,pred}\left(t\right)=\sum_{i=1}^{N}{R}_{pred,i}(t)$$

16 $$\begin{array}{c}\mathrm{min}J\left({\varvec{\theta}}\right)=\sum_{s=1}^{S}\sum_{t={t}_{off}}^{T}{\left({R}_{TOT,meas}^{s,t}-{R}_{TOT,pred}^{s,t}\right)}^{2}\end{array}$$

$${\varvec{\theta}}={{\varvec{k}}}_{{\varvec{d}}}$$

where $S$ and $T$ are the numbers of sensorgrams and of time steps in a sensorgram, respectively, and ${{\varvec{k}}}_{{\varvec{d}}}$ is a column vector containing estimates of the ${k}_{d,i}$. A better estimate of the ${k}_{a,i}$ can then also be obtained using (14).

The second optimization takes the whole sensorgram into account. We first consider a bulk refractive index contribution term (${R}_{I}$) such that:

17 $$\begin{array}{c}{R}_{TOT,pred }=\left\{\begin{array}{c}\sum_{i=1}^{N}{R}_{i}+{R}_{I}, t\le {t}_{off}\\ \sum_{i=1}^{N}{R}_{i}, t>{t}_{off}\end{array}\right.\end{array}$$

${R}_{I}$ is a local offset parameter (1 per sensorgram). A first estimate for ${R}_{I}$ can be obtained by taking the difference between the observed and predicted equilibrium signals:

18 $$\begin{array}{c}{R}_{I,s}={R}_{meas,eq}-\sum_{i=1}^{N}{R}_{eq,pred,i}\end{array}$$

We then proceed by least square over whole sensorgrams:

19 $$\begin{array}{c}\mathrm{min}J\left({\varvec{\theta}}\right)=\sum_{s=1}^{S}\sum_{t=1}^{T}{\left({R}_{TOT,meas}^{s,t}-{R}_{TOT,pred}^{s,t}\right)}^{2}\end{array}$$

$${\varvec{\theta}}={\left[{{\varvec{k}}}_{{\varvec{a}}}^{\boldsymbol{^{\prime}}},{{\varvec{k}}}_{{\varvec{d}}}^{\boldsymbol{^{\prime}}} {{\varvec{R}}}_{{\varvec{m}}{\varvec{a}}{\varvec{x}}}^{\boldsymbol{^{\prime}}},{{\varvec{R}}}_{{\varvec{I}}}^{\boldsymbol{^{\prime}}} \right]}^{^{\prime}}$$

**Figure 1:**
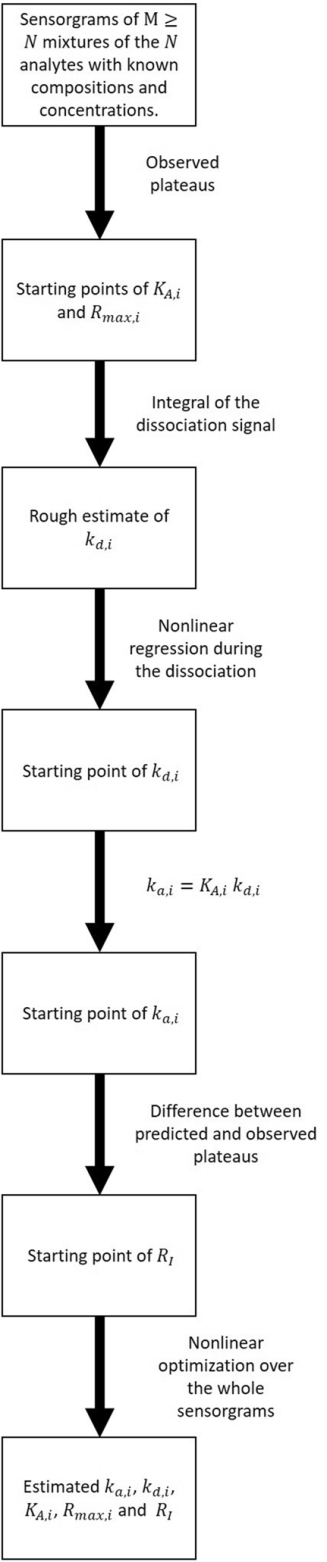
Algorithm for the estimation of the kinetic parameters of $N$ analytes. The algorithm starts by providing a legitimate starting point for a nonlinear optimization routine over the dissociation phase before a second optimization routine over the whole sensorgrams leads to the final estimates. Estimates of ${k}_{a,i}$, ${k}_{d,i}$ and ${R}_{max,i}$ for $i=1, \dots , N$ can be obtained from SPR sensorgrams of $M$ mixtures of the $N$ analytes $(M\ge N)$. The fit is said ‘global–global’ as sensorgrams corresponding to different overall concentrations and different mixtures are used simultaneously in the fitting procedure.

With ${{\varvec{k}}}_{{\varvec{a}}}$ and ${{\varvec{R}}}_{{\varvec{I}}}$ being column vectors containing the association rate constants and the refractive bulk contributions. Estimates obtained in (9) (${R}_{max,i}$), (16) (${k}_{a,i}$ and ${k}_{d,i}$) and in (18) (${R}_{I,s}$) are used as a starting point for this optimization routine. The solution to the optimization problem in (19) gives the final estimates of the kinetic parameters of the $N$ compounds. Figure 1 summarizes the kinetic parameter identification algorithm. Note that obtaining adequate starting points is critical to solve the optimization problem in Eq. (19). Otherwise, the optimization algorithm may remain trapped in a local minimum (data not shown). Evans et al.^31^ showed that the 2-analyte heterogeneous mixture model is not structurally globally identifiable. Rather, it is structurally locally identifiable, but only if the concentration of each analyte is known. This implies that it is possible to obtain a global minimum to our optimization problem, but only if the starting point for the optimization routine is adequate. The proposed algorithm can provide such starting points using a negligible amount of time (less than a minute for $N=4$) and computing capacity. One could also verify that a similar optimum is reached even if the starting point is slightly altered but remains adjacent to what is suggested by our method.

### Composition of an ${\varvec{N}}$-analyte mixture

The kinetic parameters and the ${R}_{max,i}$ obtained from a training data set can now be used to infer the composition of an unknown mixture of the same $N$ compounds. For this algorithm to be applied, the following conditions must be met:

- The ${k}_{a,i}$, ${k}_{d,i}$ and ${R}_{max,i}$ are known for the $N$ compounds.
- Sensorgrams reaching plateaus are available for the unknown mixture.
- The total concentration of all the analytes being combined (${C}_{TOT}$) is known for all sensorgrams.

The problem of identifying the composition of a mixture comes down to determining the set of ${F}_{i}$ that best fit the data. With no prior information, the starting point is chosen to be:

20 $$\begin{array}{c}{F}_{i,start}=\frac{1}{N} \forall i=1,\dots ,N\end{array}$$

A first optimization is carried out by considering only the dissociation phase, as analytical solutions can be easily obtained for this part of the sensorgrams (see (15)).

21 $$\begin{array}{c}\mathrm{min}J\left(\theta \right)=\sum_{s=1}^{S}\sum_{t={t}_{off}}^{T}{\left({R}_{TOT,meas}^{s,t}-{R}_{TOT,pred}^{s,t}\right)}^{2}\end{array}$$

$$\theta ={\varvec{F}}$$

With the following constraints:

22 $$\begin{array}{c}0\le {F}_{i}\le 1 \forall i=1,\dots ,N\end{array}$$

$$\sum_{i=1}^{N}{F}_{i}=1$$

where ${\varvec{F}}$ is a column vector containing the ${F}_{i}$. This provides coarse estimates for the ${F}_{i}$ that can be used as starting points for a second optimization routine performed over the whole sensorgrams:

23 $$\begin{array}{c}\mathrm{min}J\left(\theta \right)=\sum_{s=1}^{S}\sum_{t=1}^{T}{\left({R}_{TOT,meas}^{s,t}-{R}_{TOT,pred}^{s,t}\right)}^{2}\end{array}$$

$$\theta ={\varvec{F}}$$

**Figure 2:**
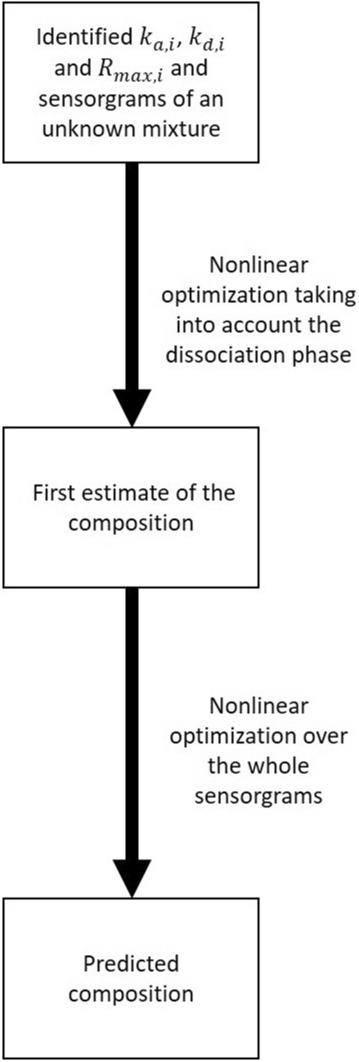
A two-step algorithm for the estimation of the composition of an unknown mixture of $N$ analytes with known kinetic parameters. The starting point for the fraction of each analyte is set to $1/N$ for the first optimization routine. This optimization uses only the dissociation phase of the sensorgrams. The results of the first step are used as the starting points of the second optimization routine. Note that the total concentration (${C}_{TOT}$) of analytes in the mixture is known, but not the mixture composition (fraction of each analyte).

With the same constraints given in (22). Note that terms accounting for the bulk refractive contributions (${R}_{I,s}$) were added for every sensorgram. These are optimized together with ${\varvec{F}}$ in (23). Initial estimates for these parameters can be obtained using (18). Figure 2 summarizes the composition identification algorithm.

### Confidence intervals on the kinetic parameters

The confidence intervals can be computed based on the standard error^7,9^. For the ${k}^{th}$ parameter, the standard error is given by:

24 $$\begin{array}{c}SE\left(k\right)=\sqrt{{\left[{{\varvec{H}}}^{-1}\right]}_{k,k} {\chi }^{2}}\end{array}$$

where ${\varvec{H}}$ is the hessian matrix and ${\left[{{\varvec{H}}}^{-1}\right]}_{k,k}$ corresponds to the ${k}^{th}$ element on the diagonal of the inverse of ${\varvec{H}}$. ${\chi }^{2}$, which depends on the number of estimated parameters ($p=3N+S$) and on the number of data points ($n=ST$), is calculated as follows:

25 $$\begin{array}{c}{\chi }^{2}=\frac{\sum_{s=1}^{S}\sum_{t=1}^{T}{\left({R}_{TOT,meas}^{s,t}-{R}_{TOT,pred}^{s,t}\right)}^{2}}{n-p}\end{array}$$

Details on the computation of the hessian matrix are given in Supplementary Appendix A. Once the standard error of a given parameter has been computed, a $100\left(1-\alpha \right)\%$ confidence interval can be obtained using Student’s T distribution:

26 $$\begin{array}{c}k={k}_{pred}\pm {t}_{n-p,\alpha /2}*SE\left(k\right)\end{array}$$

### Confidence intervals on the fractions

Because the optimization problems for mixture composition estimation involve inequality constraints (all fractions must be between 0 and 1), it is necessary to obtain non-symmetrical confidence intervals for the fractions. We have applied a method based on the Fisher $F$ statistic^32^. Considering the optimization problem given in (23), we have:

27 $$\begin{array}{c}\frac{{\left.J\left({\varvec{\theta}}\right)\right|}_{{F}_{i}={F}_{i0}}-J\left(\widehat{{\varvec{\theta}}}\right)}{J\left(\widehat{{\varvec{\theta}}}\right)/\left(n-p\right)}\sim F\left(1,n-p\right)\end{array}$$

With $J\left(\widehat{{\varvec{\theta}}}\right)$ being the value of the objective function at the optimum point (estimated fractions) and ${\left.J\left({\varvec{\theta}}\right)\right|}_{{F}_{i}={F}_{i0}}$ the value of the objective value obtained by optimizing with an added constraint ${F}_{i}={F}_{i0}$. The boundaries of the $100\left(1-\alpha \right)\%$ confidence interval are such that:

28 $$\begin{array}{c}{\left.J\left(\theta \right)\right|}_{{F}_{i}={F}_{i0}}-J\left(\widehat{\theta }\right)={F}_{1-\alpha }\left(1,n-p\right)*\frac{J\left(\widehat{\theta }\right)}{n-p}\end{array}$$

${F}_{1-\alpha }$ is a quantile of the Fisher law with the appropriate number of degrees of freedom. The method consists in changing each ${F}_{i}$ progressively (to ${F}_{i}^{^{\prime}}$) starting from its estimated value. The optimization is performed with the added constraint ${F}_{i}={F}_{i}^{^{\prime}}$. If the condition in (28) is met, ${F}_{i}^{^{\prime}}$ corresponds to ${F}_{i0}$, which is the boundary of the confidence interval of ${F}_{i}$. Otherwise, a bigger disturbance needs to be applied. By disturbing in turn each ${F}_{i}$ positively and negatively, we can obtain non-symmetrical confidence bounds of the estimated fractions. More details on the algorithm are provided in Supplementary Appendix B.

## Results

For the purpose of testing our data analysis algorithms for multiple-analyte SPR injections, carbonic anhydrase II (CAII) was immobilized at the surface of a SPR biosensor. CAII was selected because its interactions with many small molecular compounds have been extensively characterized by SPR. Among known and well-characterized CAII binders, CBS, BDS, sulfanilamide and furosemide were selected because they encompass a broad range of kinetic constants and refractive index increments (RII)^12^. In order to account for these RII discrepancies, our model considers an individual ${R}_{max,i}$ for each analyte.

**Figure 3:**
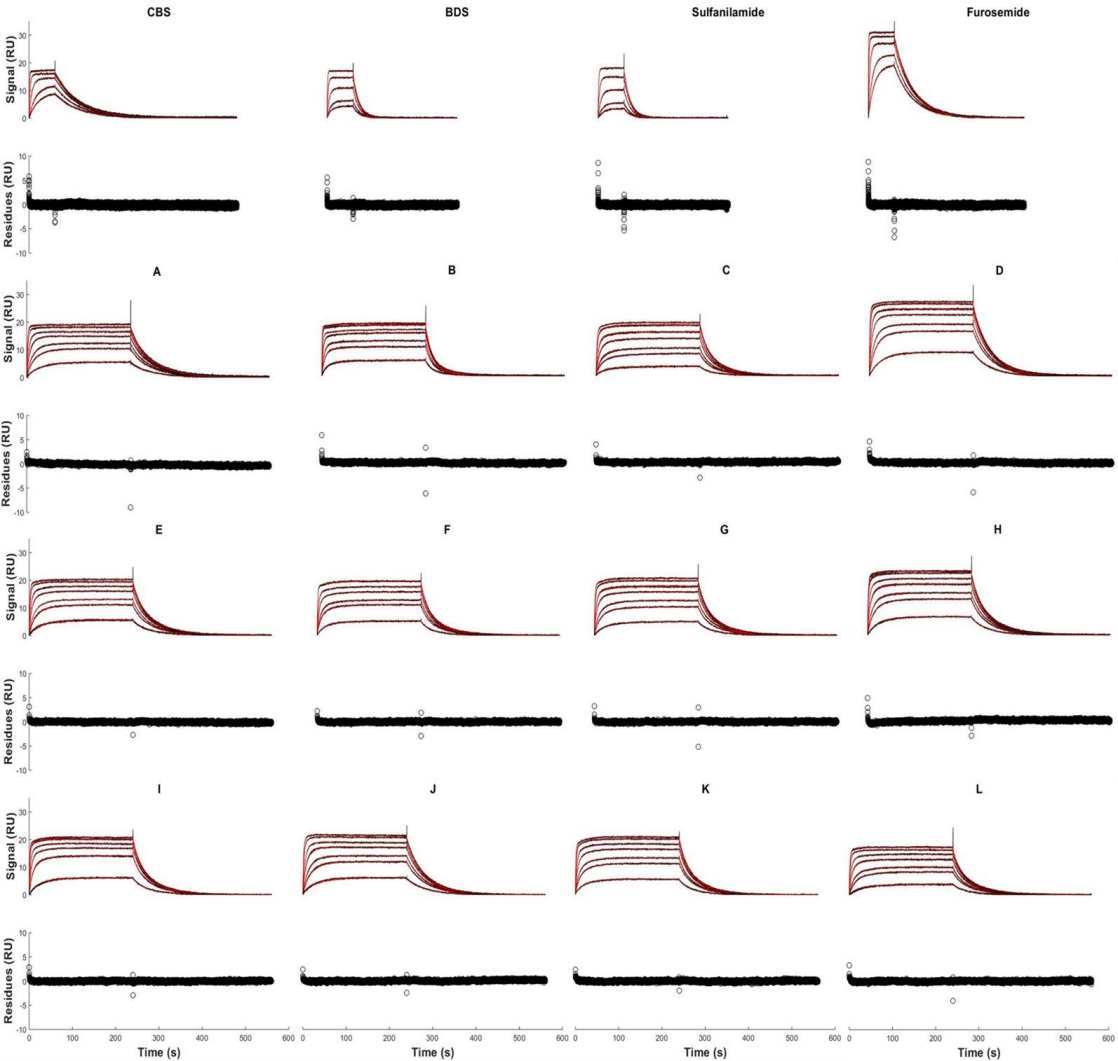
Kinetic analysis of the injection of the 4 compounds and the 12 mixtures of compounds. Black dots correspond to control-corrected and double referenced sensorgrams for all experiments. Red lines correspond to global fits for the single-analyte experiments (CBS, BDS, sulfanilamide and furosemide) and to ‘global–global’ fits for each data set for 70–10–10–10 mixtures (A to D, see Table 1 for exact composition), 40–20–20–20 mixtures (E to H, see Table 1 for exact composition) and random proportion mixtures (I to L, see Table 1 for exact composition). For the single-analyte experiments, the concentrations of CBS, BDS, sulfanilamide and furosemide varied respectively from 1.06 μM to 52.98 μM, 210 nM to 10.49 μM, 1.01 μM to 50.27 μM and 1.01 μM to 50.52 μM. The total concentrations (${C}_{TOT}$) of the mixtures varied from 300 nM to 15 μM for all mixtures. Here, the composition of each mixture is assumed to be known. The related residual plot is given below each sensorgram data set. This figure was generated with the Matlab R2018b software platform (The Mathworks, Natick, USA, www.mathworks.com/products/matlab.html).

A total of 16 SPR experiments were performed. The 4 compounds were first injected alone at 5 different concentrations, then 12 mixtures all containing the 4 compounds were injected at 7 different total concentrations. To determine if it is possible to extract reliable kinetic parameters from mixtures of analytes at any molar ratio, various sets of experiments were performed. First, we injected mixtures in which one of the analytes is dominant (i.e. 70% on a molar basis) while the other 3 analytes account for 10% each (mixtures A to D, Table 1). Second, to investigate whether mixtures of lesser purity would also allow to extract kinetics, each analyte was injected at a proportion of 40% while the others completed the mix (20% each, mixtures E to H, Table 1). Third, 4 mixtures (I to L, Table 1) were created with random compositions to determine if a non-orthogonal mixture set could lead to accurate kinetic estimations. Sensorgrams corresponding to these 16 experiments are shown in Fig. 3. Sensorgrams corresponding to single-analyte experiments (CBS, BDS, sulfanilamide and furosemide) were globally analyzed with a 1:1 Langmuir model. The three sets of sensorgrams obtained for analyte mixtures (i.e. those shown in panels A–D, E–H and I–L) were fitted assuming that (1) each mixture composition was known and (2) each analyte within each mixture competed to bind to CAII according to a 1:1 Langmuir model. In these cases, the fits can be said to be ‘global–global’ because sensorgrams of different concentrations and different mixtures are simultaneously analyzed to extract the kinetic parameters. In all cases, to mimic an automated data preparation routine, data were control-corrected without excluding any data point prior to fitting. This is reflected by the presence of spikes at the beginning of each injection (i.e., analyte and buffer) phase in Fig. 3. The fits are otherwise excellent (${\chi }^{2}$ in the order of 0.1 for all data sets) with no prevalent trends in the residuals (see residual plots, Fig. 3).

**Figure 4:**
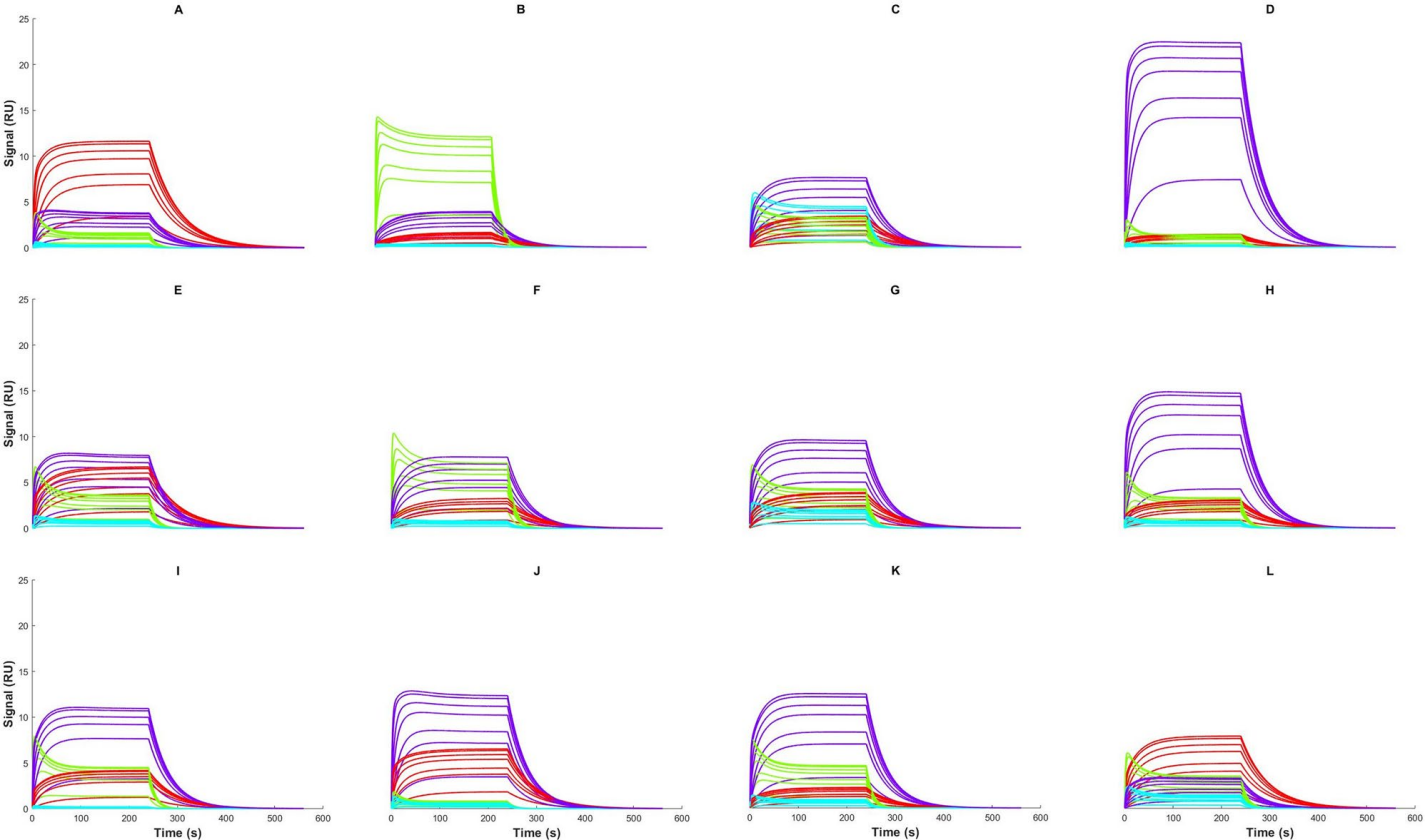
Kinetic analysis of the contribution of CBS (red), BDS (green), sulfanilamide (cyan) and furosemide (purple) to the SPR response of each mixture. The kinetic parameters identified from each data set (see Table 2) were used to obtain the contributions to related sensorgrams. The sum of these contributions gives the ‘global–global’ fits shown in Fig. 3, minus the bulk refractive index contribution ${R}_{I}$. The total concentrations (${C}_{TOT}$) ranged from 300 nM to 15 μM for all mixtures. This figure was generated with the Matlab R2018b software platform (The Mathworks, Natick, USA, www.mathworks.com/products/matlab.html).

Table 2 shows the estimated kinetic parameter values and their 95% confidence intervals as well as the estimated affinity of each analyte for each fit. The width of the confidence intervals is less than 5% of the estimated parameter value for most cases. Using these estimated parameters and Eq. (2), it is possible to compute the contribution of each analyte to the SPR response. The contribution of each analyte is shown in Fig. 4 to facilitate the understanding of the competitive phenomena occurring at the surface during the multiple-analyte injection phase. Of considerable interest, BDS, the analyte with the highest association rate (${k}_{a}$) always binds to CAII first, before being progressively replaced by an analyte with slower kinetics but higher affinity for CAII (i.e., furosemide) or by analytes present in higher proportions in the injected mixture. Sulfanilamide, which has the lowest affinity for CAII among the analytes we used, has small to almost negligible contributions to the response in most mixtures, except for mixtures C and G where it is present in a dominating proportion (see Table 1).

**Table 2:** Kinetic parameters identified from single- and multiple-analyte injections of mixtures of known composition. 95% confidence interval are given in parentheses.

Data set	Compound	${{\varvec{k}}}_{{\varvec{a}}}\boldsymbol{ }\left({{10}^{4}{\varvec{s}}}^{-1}{{\varvec{M}}}^{-1}\right)$	${{\varvec{k}}}_{{\varvec{d}}}\boldsymbol{ }\left({{10}^{-3}{\varvec{s}}}^{-1}\right)$	${{\varvec{R}}}_{{\varvec{m}}{\varvec{a}}{\varvec{x}}}\boldsymbol{ }\left({\varvec{R}}{\varvec{U}}\right)$	${{\varvec{K}}}_{{\varvec{A}}}\boldsymbol{ }\left({10}^{6}{{\varvec{M}}}^{-1}\right)$
Single-analyte	CBS	2.16 (2.15; 2.16)	17.5 (17.4; 17.5)	16.42 (16.41; 16.44)	1.23
	BDS	10.02 (9.98; 10.06)	70.1 (69.9; 70.2)	17.01 (16.98; 17.04)	1.43
	Sulfanilamide	1.41 (1.41; 1.42)	66.8 (66.7; 67.0)	18.68 (18.65; 18.72)	0.21
	Furosemide	4.22 (4.21; 4.22)	23.0 (23.0; 23.1)	29.21 (29.20; 29.23)	1.83
70–10–10–10 mixtures	CBS	2.36 (2.35; 2.37)	17.2 (17.1; 17.2)	16.42 (16.40; 16.45)	1.37
	BDS	9.41 (9.37; 9.46)	71.8 (71.4; 72.2)	17.24 (17.19; 17.30)	1.31
	Sulfanilamide	1.44 (1.43; 1.46)	62.5 (61.5; 63.5)	18.40 (18.24; 18.56)	0.23
	Furosemide	4.13 (4.11; 4.14)	23.0 (23.0; 23.1)	28.63 (28.59; 28.66)	1.80
40–20–20–20 mixtures	CBS	1.96 (1.93; 1.99)	16.5 (16.4; 16.6)	16.97 (16.93; 17.02)	1.19
	BDS	9.86 (9.79; 9.93)	73.0 (72.2; 73.8)	16.40 (16.30; 16.50)	1.35
	Sulfanilamide	1.63 (1.58; 1.67)	60.5 (57.6; 63.3)	18.83 (18.40; 19.26)	0.27
	Furosemide	4.17 (4.14; 4.20)	23.7 (23.7; 23.8)	27.86 (27.78; 27.95)	1.76
Random mixtures	CBS	2.22 (2.19; 2.24)	18.2 (18.2; 18.3)	15.89 (15.85; 15.92)	1.22
	BDS	9.25 (9.17; 9.32)	77.1 (76.1; 78.1)	16.00 (15.91; 16.08)	1.20
	Sulfanilamide	2.05 (1.98; 2.11)	72.8 (68.9; 76.6)	14.92 (14.61; 15.24)	0.28
	Furosemide	4.22 (4.19; 4.25)	24.6 (24.5; 24.6)	27.64 (27.55; 27.73)	1.72

The estimated affinity (${K}_{A}$) is also reported.

### Is it possible to extract kinetic parameters from a mixture of analytes?

Figure 3 showed good fits between the calculated and observed SPR responses. In order to evaluate the accuracy of the kinetic parameter determination when the parameters are extracted from mixtures of analytes, we compared them to the kinetic parameters determined from single-analyte experiments. For comparison sake, the deviation (or relative error—see Table 3) was calculated as follows for a given parameter $k$:

**Table 3:** Deviation between the parameters identified from single-analyte experiments and parameters identified by fitting the multi-analyte model independently to the 3 data sets (70–10–10–10, 40–20–20–20 and random mixtures).

Data set	Compound	${{\varvec{k}}}_{{\varvec{a}}}\boldsymbol{ }\left(\boldsymbol{\%}\right)$	${{\varvec{k}}}_{{\varvec{d}}}\boldsymbol{ }\left(\boldsymbol{\%}\right)$	${{\varvec{R}}}_{{\varvec{m}}{\varvec{a}}{\varvec{x}}}\boldsymbol{ }\left(\boldsymbol{\%}\right)$	${{\varvec{K}}}_{{\varvec{A}}}\boldsymbol{ }\left(\boldsymbol{\%}\right)$
70–10–10–10 mixtures	CBS	9.3	1.7	0.0	10.0
	BDS	6.1	2.4	1.4	9.1
	Sulfanilamide	2.1	6.4	1.5	8.4
	Furosemide	2.1	0.0	2.0	2.2
40–20–20–20 mixtures	CBS	9.3	5.7	3.4	3.9
	BDS	1.6	4.1	3.6	5.8
	Sulfanilamide	15.6	9.4	0.8	21.7
	Furosemide	1.2	3.0	4.6	4.3
Random mixtures	CBS	2.8	4.0	3.2	1.2
	BDS	7.7	10.0	5.9	19.1
	Sulfanilamide	45.4	9.0	20.1	25.0
	Furosemide	0.0	7.0	5.4	7.0

$$deviation\left(\%\right)=\frac{\left|{k}_{single}-{k}_{multi}\right|}{{k}_{single}}*100$$

The deviation between parameters obtained with single and multiple-analyte experiments should ideally be close to 0. The deviations for the 70–10–10–10 data set (mixtures A to D) were of the same magnitude as the repeatability errors. Those were evaluated by performing 3 independent single-analyte experiments using the same stock solution of CBS and the same CAII surface and ranged from 0 to 10% (data not shown). The error in the estimation of the kinetic parameters made by the fitting algorithm is indiscernible from the experimental error made by producing the 4 independent mixtures of analytes, thus indicating that the fitting algorithm was able to properly extract the kinetic parameters of the 4 analytes using the 70–10–10–10 data set.

### How pure do the mixtures need to be?

The limits of the approach were then tested with mixtures of lesser purity (the 40–20–20–20 data set, mixtures E to H). Once again, the estimated parameters were very close to those obtained by single-analyte experiments for 3 of the analytes (Table 3). A bigger deviation was observed for sulfanilamide. This can be attributed to its low affinity for CAII, which leads to a limited or negligible contribution in the sensorgrams corresponding to the 40–20–20–20 mixtures data set (see plots E–H in Fig. 4). The goodness of fit of the model is then less sensitive to the quality of the estimated parameters of sulfanilamide. This leads to wider confidence intervals, especially for sulfanilamide (see Table 2), thus complicating the extraction of the kinetic parameters of this analyte. Nonetheless, our results indicate that reliable parameters could be obtained for CBS, BDS and furosemide even with mixtures of a lower purity.

### Can a non-orthogonal set of mixtures be used?

We then tested the method with a non-orthogonal mixture set. Here, ‘non-orthogonal’ refers to the fact that the ${\varvec{F}}$ matrix is not orthogonal (see Eqs. 7 and 8), although it needs to remain invertible. The compositions of the mixtures within this data set are given in Table 1 (Random mixtures data set, mixtures I–L). One could expect that mixtures for which none of the analytes has a dominating proportion would lead to confounding the effects of the different analytes, and hence poor estimates. However, Table 3 shows that this is most likely not an issue, as small deviations were observed for CBS, BDS and furosemide. Only the estimates for sulfanilamide deviated significantly, more likely because of the small contribution of sulfanilamide to the sensorgrams in the data set (see plots I to L in Fig. 4).

We validated that our algorithm can extract the kinetic parameters of a group of analytes from sensorgrams corresponding to various mixtures of these analytes. The main limitation is that each analyte must have a non-negligible contribution to the recorded SPR response in at least one of the mixtures being fitted. Failure to comply with this requirement leads to bigger estimation errors (especially for the analyte that contributes the less), and to larger confidence intervals. Better estimates (i.e. smaller deviations, narrower confidence intervals) are obtained when low-affinity compounds are present in higher proportion in at least one mixture of the data set being fitted.

### Can we estimate the composition of a mixture of analytes?

**Figure 5:**
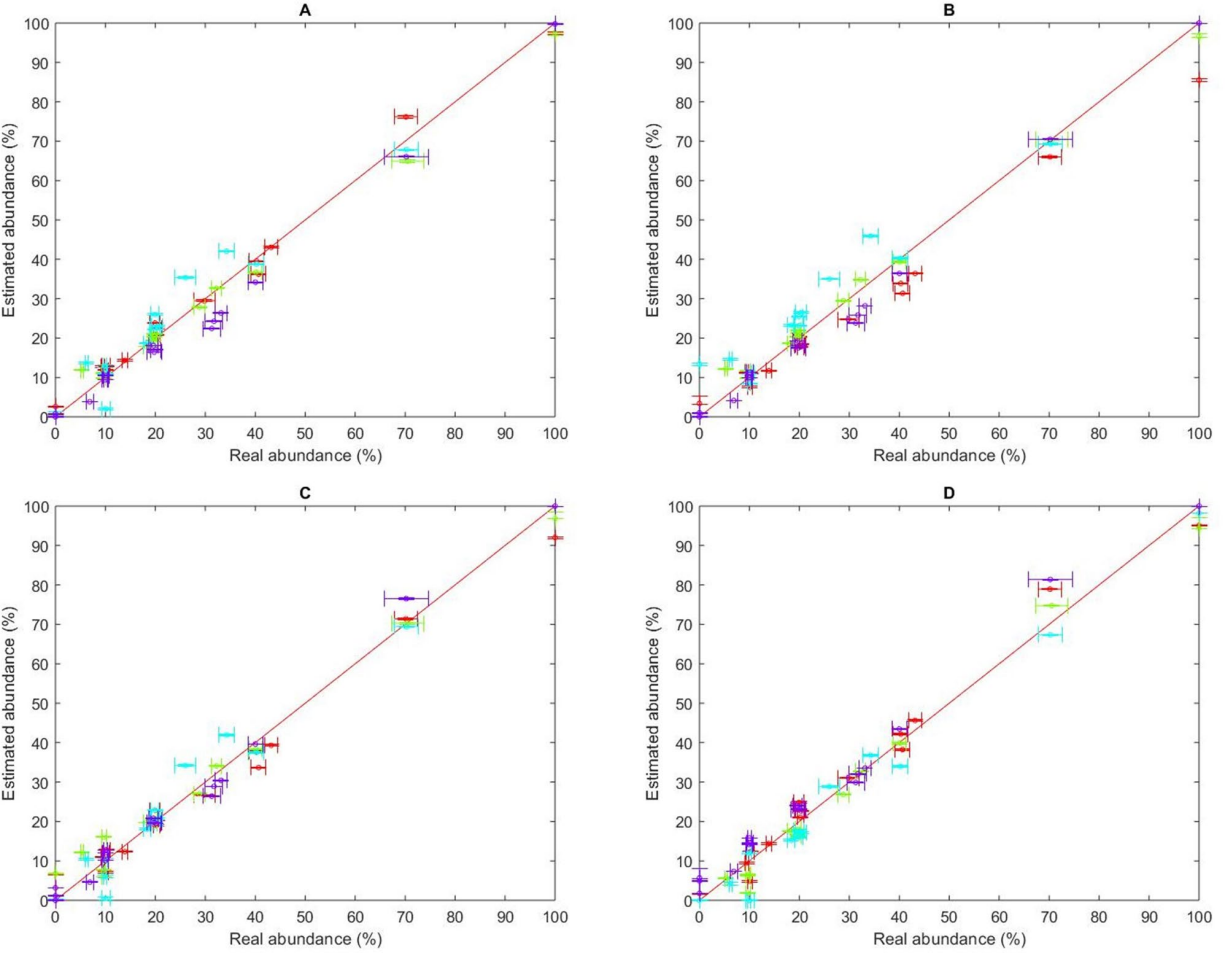
Estimated fractions of CBS (red), BDS (green), sulfanilamide (cyan) and furosemide (purple) with respect to the true fraction values in each of the 16 mixtures. The fractions were estimated using the kinetic parameters and ${R}_{max,i}$ identified from different data sets, corresponding to (**A**) Single-analyte injections; (**B**) 70–10–10–10 mixtures (mixtures A to D); (**C**) 40–20–20–20 mixtures (mixtures E to H); (**D**) Random mixtures (mixtures I to L). The horizontal error bars were obtained by propagating the systematic error of the pipettes (Pipetman Neo P10, P20, P200 and P1000 and Microman M1000E) and the balance (Dever Instrument Company AA-160) that were used to conduct the experiments. The vertical error bars correspond to 95% confidence intervals computed using the Fisher F statistic. Here, we assumed that the total concentration of all analytes (${C}_{TOT}$) is known, but the composition of each mixture (fraction of each analyte) is not. This figure was generated with the Matlab R2018b software platform (The Mathworks, Natick, USA, www.mathworks.com/products/matlab.html).

We then explored if prior knowledge of the kinetic parameters of each analyte could be used to infer the composition of each mixture of analytes. Using a given set of kinetic parameters we previously determined (see Table 2), we estimated the composition of all 16 mixtures. Figure 5 compares the estimated fractions to the actual ones. Note that vertical error bars on Fig. 5 correspond to the 95% confidence bounds for the estimated fractions and horizontal bars represent the uncertainty on the real fractions. The latter comes from the propagation of systematic instrument errors during weighting and pipetting. Of considerable interest, prior knowledge of the kinetic parameters, as estimated from any data set, allowed us to predict the compositions of every mixture with good accuracy.

**Figure 6:**
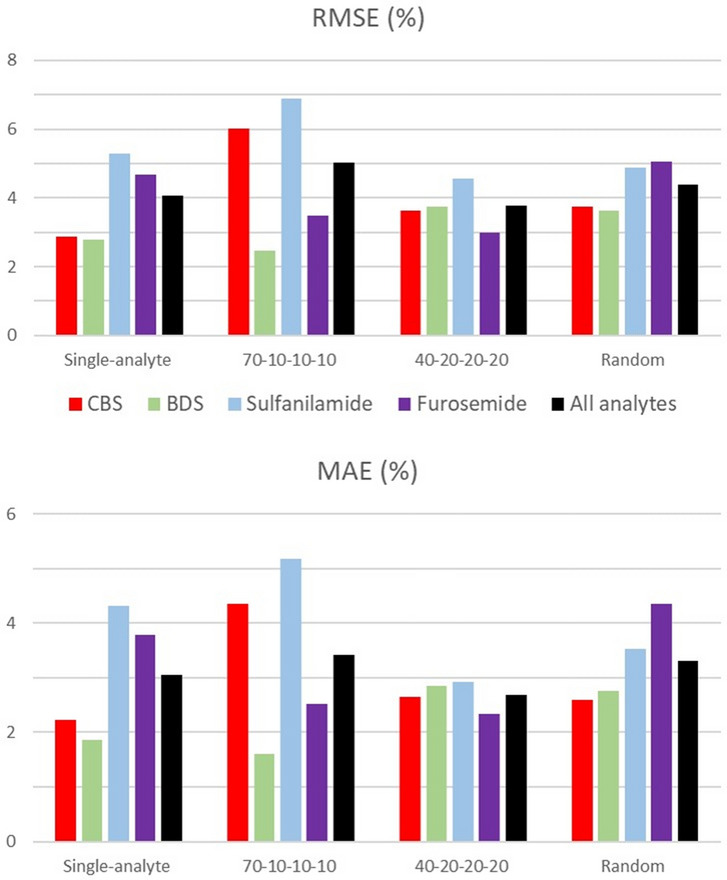
Residual mean square error (RMSE, top) and mean absolute error (MAE, bottom) calculation. The composition of each mixture was estimated using the kinetic parameters and ${R}_{max,i}$ values identified from different data sets (single-analyte, 70–10–10–10 mixtures, 40–20–20–20 mixtures and random mixtures). The estimated composition was compared to the actual composition of the mixtures outside of the data set used to identify the kinetic parameters. The residual mean square error (RMSE, top) and the mean absolute error (MAE, bottom) were calculated on an analyte basis for CBS, BDS, sulfanilamide and furosemide and by considering all the estimates at once (all analytes). This procedure was repeated for all data sets.

To investigate whether prior knowledge of the kinetic parameters, as estimated from a given data set, led to more accurate fraction estimates, we computed the Root Mean Square Error (RMSE) between the estimated and actual fractions independently for each set of kinetic parameters (see Fig. 6). The RMSE can be strongly influenced by the presence of a few large errors, so we also computed the Mean Absolute Error (MAE), which can temper their contribution^33,34^.

We computed the mean prediction error for one analyte at a time and then for all the analytes at once. Results are shown in Fig. 6. For a given set of parameters, only the mixtures which were not used to identify the kinetic parameters were used to compute the mean error (each set of kinetic parameters was thus identified using 4 mixtures and tested across 12 other mixtures).

Figure 6 shows similar trends for the RMSE and the MAE. No apparent difference is perceived between the performance of the different sets of kinetic parameters in predicting the mixture compositions when considering all the compounds. This is of considerable interest as it indicates that, even when using mixtures of a lesser purity to identify the kinetic parameters (which leads to larger deviations in the estimated kinetic parameters—see Table 3), mixture compositions can still be estimated with similar accuracy. All sets of kinetic parameters except the one obtained with random mixtures led to larger errors in estimating the fraction of sulfanilamide. This is because of its low affinity to CAII and therefore small contribution to the recorded SPR response.

## Discussion

An algorithm for the identification of the binding kinetics of multiple analytes from the SPR sensorgrams corresponding to mixtures of these analytes has been proposed (Fig. 1). Furthermore, we postulated that, using the identified kinetics, the composition of other mixtures of the same analytes could be estimated (Fig. 2). As a proof of concept, the binding kinetics of four small molecular-weight compounds (CBS, BDS, sulfanilamide and furosemide) to the enzyme CAII were studied. The kinetic parameters derived from classical single-analyte experiments (analyzed via standard numerical methods) and the kinetic parameters derived from multiple-analyte experiments (Fig. 3) analyzed using our algorithm were shown to match. This remained true even when mixtures of lower purity were used for the identification (Table 3). Additionally, the kinetic parameters obtained from one data set could be used to estimate the compositions of the other mixtures with excellent accuracy (Figs. 5, 6). Indeed, we generated 16 mixtures of 4 analytes, of which 4 were used for training (identification of the kinetic parameters), while the remainder was used for validation (estimating the composition of the other 12 mixtures). We pushed the study further by performing 3 other rounds of study in which the 4 mixtures used for training were changed and the remaining 12 were used for validation. For example, we identified kinetic parameters using mixtures A–B–C–D (as reported in Table 2), then used the estimated parameters obtained with mixtures A–B–C–D to estimate the composition of mixtures E–F–G–H–I–J–K–L and pure solutions (Fig. 5). With this approach, we were able to show that our algorithm was extremely robust since, no matter which data set were used for training, validation was good, or even excellent, with the other mixtures (Fig. 6).

In contrast to the previous body of work we proposed on kinetic parameter identification^10,12^ or concentration determination^20^, the goal of the algorithms presented here is not to increase the throughput of SPR-based experiments. Indeed, for the identification of the kinetic parameters of $N$ analytes, our approach requires $N$ sets of sensorgrams, as for a classical single-analyte approach. Rather, we here demonstrate that for mixtures of macromolecules that are hard to purify, one may determine the kinetic parameters of each individual binder without any further purification. For instance, it is well-known that immunoglobulin G (IgG) production in bioreactors typically results in a heterogeneous distribution of glycoforms of the same IgG^26^, in turn impacting the IgG interactions with their Fcγ receptors, and the IgG therapeutic efficacy^21–27^. Hence, an algorithm similar to ours could potentially uncover meaningful binding kinetics of different IgG glycoforms without having access to pure IgG glycoform solutions. To uncover the kinetics of $N$ different glycoforms, one would require $N$ (potentially heterogeneous in terms of glycoforms) different samples with different known compositions (relative abundance of the glycoforms). Producing $N$ differently glycosylated samples can be achieved by varying the cell culture conditions or through the use of various cellular and protein engineering strategies^35^. The compositions could be determined via mass spectroscopy, as it has already been performed in^21–27^. Once the glycoform kinetics have been uncovered, unknown glycoform mixtures could then be characterized.

The absence of mass transport limitations (MTL)^36,37^ and the Langmuirian behavior of each analyte/ligand interaction we assumed was verified for the model system we used in this study (CAII/small molecular weight drugs). However, these assumptions may not always apply. It may thus be necessary to adjust the model to other mechanisms of binding so as to fit the experimental data. In the case of MTL, as plateau values of the sensorgrams would remain the same^36,37^, the determination of the equilibrium constants (${K}_{A}$) would still be possible with our approach.

High molecular weight analytes, such as proteins, usually have similar refractive index increments (RII), thus implying that the ratio of their respective ${R}_{max,i}$ should be proportional to the ratio of their molecular weights^10,15,38–40^. Knowledge of the molecular weights of every analyte would then reduce the number of parameters to optimize using our strategy, as including only one ${R}_{max}$ value would be sufficient. In contrast, small molecular weight compounds often exhibit varying RII. Such is the case for those used in the present study^12^. Thus, it was necessary to account for one ${R}_{max,i}$ per analyte.

We found an affinity between sulfanilamide and CAII that is almost an order of magnitude lower than that of the other compounds tested. An analyte with a considerably lower affinity for the ligand will often have a negligible contribution to the SPR response, especially in mixtures where it is not present at a high purity. This presumably explains why bigger deviations were observed in the identified kinetic parameters of sulfanilamide. Larger errors were also made for sulfanilamide when estimating mixture compositions. This phenomenon could be even more noticeable in systems where two or more analytes are found to have considerably lower affinities than the rest of the analytes. In such a system, the kinetic parameters identified for the low-affinity analytes could exhibit significant deviations and large confidence intervals. When possible, a mixture containing a high purity of at least one of these analytes should be added to the training data to facilitate the kinetic parameter identification process. If such a thing is not possible, we suggest pooling low affinity analytes together, i.e. treating two or more analytes as a single one. Pooling analytes with very similar kinetics could also be preferable, as it might be difficult to distinguish their respective effect on the SPR response. The kinetics obtained would then be slightly biased, but still usable to partially characterise an unknown mixture.

It is important to note that the experiments in this study aimed at validating the method and were not intended to be optimal in terms of experimental time and material consumption. Notably, the analyte injection time was kept very long to ensure that equilibrium was reached. This resulted in longer sensorgrams with more data points, in turn narrowing the confidence intervals on the kinetic parameters and decreasing uncertainties on the estimated compositions. Also, the number of sensorgrams used (7 duplicates per mixture) was intended to survey the experimental space (from a low concentration that gives a minimal observable response to a high concentration that is close to saturation of the surface). However, this may not be optimal and previous studies with single-analyte injections have shown that using a lower number of injections does not always result in larger parameter uncertainty^7^. One may thus minimize experimental time and material consumption necessary to apply our strategy in a similar fashion as performed for single-analyte^9^ and for two-analyte experiments^10^.

## Conclusion

We suggested a framework for the analysis of multiple-analyte injections over an SPR biosensor surface. The proposed algorithms were shown robust and efficient to extract the kinetics of at least four analytes, as well as to use these parameters to estimate the composition of unknown mixtures from their SPR sensorgrams. The limitations of the framework are its simple 1:1 competitive Langmuir binding scheme and the fact that knowledge of the total concentration of all analytes combined is required. However, the total concentration could be added as a variable to optimize when characterizing an unknown mixture. Our framework broadens the application of SPR biosensing to the detailed characterization of complex analyte mixtures, particularly in contexts where separating the constituents is challenging or impossible. Given the increased use of SPR biosensing, especially in the field of drug discovery and development, the findings of this study may be of interest to a broad community in both academia and industry.

## Supplementary Information

Supplementary Information.
