# Supplementary material for: Determination of the composition of heterogeneous binder solutions by surface plasmon resonance biosensing
Source: Sci Rep. 2021 Feb 11;11:3685. doi: 10.1038/s41598-021-83268-z (PMC7878517; doi:10.1038/s41598-021-83268-z)
Supplement: Supplementary file 1 — Supplementary Information. [file 41598_2021_83268_MOESM1_ESM.pdf]

Title

Determination of the composition of heterogeneous binder solutions by surface plasmon resonance biosensing

Authors

Jimmy Gaudreault, Benoît Liberelle, Yves Durocher, Olivier Henry*, Gregory De Crescenzo*

*: These authors contributed equally to this work.

1. Supplementary Appendix A - Hessian matrix with N analytes

The hessian matrix can be approximated by:

$$H \approx \sum_{s=1}^{S}\sum_{t=1}^{T}\left(\frac{\partial R_{TOT,pred}^{s,t}}{\partial \boldsymbol{\theta}}\right)^{T}\left(\frac{\partial R_{TOT,pred}^{s,t}}{\partial \boldsymbol{\theta}}\right)$$

$$\boldsymbol{\theta} = [\boldsymbol{k_a'}, \boldsymbol{k_d'}\ \boldsymbol{R_{max}'}, \boldsymbol{R_I'}\ ]'$$

With

$$\frac{\partial R_{TOT,pred}}{\partial \boldsymbol{\theta}} = \sum_{i=1}^{N}\frac{\partial R_{pred,i}}{\partial \boldsymbol{\theta}}$$

The derivatives in (31) can be evaluated by solving the following ODEs along with the system of ODE in (6):

$$\frac{d\boldsymbol{R}}{dt} = f(\boldsymbol{R},\boldsymbol{\theta})$$

$$\frac{d}{dt}\frac{d\boldsymbol{R}}{d\boldsymbol{\theta}} = \frac{\partial \boldsymbol{f}}{\partial \boldsymbol{\theta}} + \frac{\partial \boldsymbol{f}}{\partial \boldsymbol{R}}\frac{\partial \boldsymbol{R}}{\partial \boldsymbol{\theta}}$$

$$\boldsymbol{R}(0) = [R_1(0), \dots R_N(0)]' = [0,\cdots,0]'$$

$$\left.\frac{\partial \boldsymbol{R}}{\partial \boldsymbol{\theta}}\right|_{t=0} = 0$$

$$f_i = k_{ai}F_iC_{TOT}R_{max,i}\left(1 - \sum_{j=1}^{N}\frac{R_j}{R_{max,j}}\right) - k_{di}R_i \quad \forall\, i = 1,\dots,N$$

Here we present a way to compute the necessary gradient $\frac{\partial R_{TOT,pred}}{\partial \boldsymbol{\theta}}$ to compute the hessian matrix related to the estimation of the kinetic parameters. Consider 3 matrices:

$$\boldsymbol{X_1} = \begin{bmatrix} \frac{\partial R_1}{\partial k_{a,1}} & \cdots & \frac{\partial R_1}{\partial k_{a,N}} \\ \vdots & \ddots & \vdots \\ \frac{\partial R_N}{\partial k_{a,1}} & \cdots & \frac{\partial R_N}{\partial k_{a,N}} \end{bmatrix} = \frac{\partial \boldsymbol{R}}{\partial \boldsymbol{k_a}}$$

$$\boldsymbol{X_2} = \begin{bmatrix} \frac{\partial R_1}{\partial k_{d,1}} & \cdots & \frac{\partial R_1}{\partial k_{d,N}} \\ \vdots & \ddots & \vdots \\ \frac{\partial R_N}{\partial k_{d,1}} & \cdots & \frac{\partial R_N}{\partial k_{d,N}} \end{bmatrix} = \frac{\partial \boldsymbol{R}}{\partial \boldsymbol{k_d}}$$

$$\boldsymbol{X_3} = \begin{bmatrix} \frac{\partial R_1}{\partial R_{max,1}} & \cdots & \frac{\partial R_1}{\partial R_{max,N}} \\ \vdots & \ddots & \vdots \\ \frac{\partial R_N}{\partial R_{max,1}} & \cdots & \frac{\partial R_N}{\partial R_{max,N}} \end{bmatrix} = \frac{\partial \boldsymbol{R}}{\partial \boldsymbol{R_{max}}}$$

With $\boldsymbol{R} = [R_1, \cdots R_N]'$.

Sensibility with respect to $\boldsymbol{k_{a,i}}$:

$$\frac{d\boldsymbol{X_1}}{dt}(i,i) = \frac{\partial f_i}{\partial k_{a,i}} + \frac{\partial f_i}{\partial R_i} * \frac{\partial R_i}{\partial k_{a,i}} + \sum_{j \neq i}^{N} \frac{\partial f_i}{\partial R_j} * \frac{\partial R_j}{\partial k_{a,i}}$$

$$= \frac{\partial f_i}{\partial k_{a,i}} + \frac{\partial f_i}{\partial R_i} * X_1(i,i) + \sum_{j \neq i}^{N} \frac{\partial f_i}{\partial R_j} * X_1(j,i)$$

$$\frac{d\boldsymbol{X_1}}{dt}(i,j) = \frac{\partial f_i}{\partial k_{a,j}} + \frac{\partial f_i}{\partial R_i} * \frac{\partial R_i}{\partial k_{a,j}} + \sum_{k \neq i}^{N} \frac{\partial f_i}{\partial R_k} * \frac{\partial R_k}{\partial k_{a,j}}$$

$$= \frac{\partial f_i}{\partial k_{a,j}} + \frac{\partial f_i}{\partial R_i} * X_1(i,j) + \sum_{k \neq i}^{N} \frac{\partial f_i}{\partial R_k} * X_1(k,j)$$

With partial derivatives:

$$\frac{\partial f_i}{\partial R_i} = -k_{a,i} F_i C_{TOT} - k_{d,i}$$

$$\frac{\partial f_i}{\partial R_j} = -k_{a,i} F_i C_{TOT} \frac{R_{max,i}}{R_{max,j}}$$

$$\frac{\partial f_i}{\partial k_{a,i}} = F_i C_{TOT} R_{max,i} \left(1 - \sum_{j=1}^{N} \frac{R_j}{R_{max,j}} \right)$$

$$\frac{\partial f_i}{\partial k_{a,j}} = 0$$

We obtain:

$$\frac{\partial R_{PRED,TOT}}{\partial \boldsymbol{k_a}} = \begin{bmatrix} \sum_{i=1}^{N} \frac{\partial R_i}{\partial k_{a1}} \\ \vdots \\ \sum_{i=1}^{N} \frac{\partial R_i}{\partial k_{aN}} \end{bmatrix} = \begin{bmatrix} \frac{\partial R_{PRED,TOT}}{\partial k_{a1}} \\ \vdots \\ \frac{\partial R_{PRED,TOT}}{\partial k_{aN}} \end{bmatrix}$$

Sensibility with respect to $\boldsymbol{k_{d,i}}$:

$$\frac{d\boldsymbol{X_2}}{dt}(i,i) = \frac{\partial f_i}{\partial k_{d,i}} + \frac{\partial f_i}{\partial R_i} * \frac{\partial R_i}{\partial k_{d,i}} + \sum_{j \neq i}^{N} \frac{\partial f_i}{\partial R_j} * \frac{\partial R_j}{\partial k_{d,i}}$$

$$= \frac{\partial f_i}{\partial k_{d,i}} + \frac{\partial f_i}{\partial R_i} * X_2(i,i) + \sum_{j \neq i}^{N} \frac{\partial f_i}{\partial R_j} * X_2(j,i)$$

$$\frac{d\boldsymbol{X_2}}{dt}(i,j) = \frac{\partial f_i}{\partial k_{d,j}} + \frac{\partial f_i}{\partial R_i} * \frac{\partial R_i}{\partial k_{d,j}} + \sum_{k \neq i}^{N} \frac{\partial f_i}{\partial R_k} * \frac{\partial R_k}{\partial k_{d,j}}$$

$$= \frac{\partial f_i}{\partial k_{d,j}} + \frac{\partial f_i}{\partial R_i} * X_2(i,j) + \sum_{k \neq i}^{N} \frac{\partial f_i}{\partial R_k} * X_2(k,j)$$

With partial derivatives:

$$\frac{\partial f_i}{\partial k_{d,i}} = -R_i$$

$$\frac{\partial f_i}{\partial k_{d,j}} = 0$$

We obtain:

$$\frac{\partial R_{PRED,TOT}}{\partial \boldsymbol{k_d}} = \begin{bmatrix} \sum_{i=1}^{N} \frac{\partial R_i}{\partial k_{d1}} \\ \vdots \\ \sum_{i=1}^{N} \frac{\partial R_i}{\partial k_{dN}} \end{bmatrix} = \begin{bmatrix} \frac{\partial R_{PRED,TOT}}{\partial k_{d1}} \\ \vdots \\ \frac{\partial R_{PRED,TOT}}{\partial k_{dN}} \end{bmatrix}$$

Sensibility with respect to $\boldsymbol{R_{max,i}}$:

$$\frac{d\boldsymbol{X_3}}{dt}(i,i) = \frac{\partial f_i}{\partial R_{max,i}} + \frac{\partial f_i}{\partial R_i} * \frac{\partial R_i}{\partial R_{max,i}} + \sum_{j\neq i}^{N} \frac{\partial f_i}{\partial R_j} * \frac{\partial R_j}{\partial R_{max,i}}$$

$$= \frac{\partial f_i}{\partial R_{max,i}} + \frac{\partial f_i}{\partial R_i} * X_3(i,i) + \sum_{j\neq i}^{N} \frac{\partial f_i}{\partial R_j} * X_3(j,i)$$

$$\frac{d\boldsymbol{X_3}}{dt}(i,j) = \frac{\partial f_i}{\partial R_{max,j}} + \frac{\partial f_i}{\partial R_i} * \frac{\partial R_i}{\partial R_{max,j}} + \sum_{k\neq i}^{N} \frac{\partial f_i}{\partial R_k} * \frac{\partial R_k}{\partial R_{max,j}}$$

$$= \frac{\partial f_i}{\partial R_{max,j}} + \frac{\partial f_i}{\partial R_i} * X_3(i,j) + \sum_{k\neq i}^{N} \frac{\partial f_i}{\partial R_k} * X_3(k,j)$$

With partial derivatives:

$$\frac{\partial f_i}{\partial R_{max,i}} = k_{a,i} F_i C_{TOT} \left(1 - \sum_{j\neq i}^{N} \frac{R_j}{R_{max,j}}\right)$$

$$\frac{\partial f_i}{\partial R_{max,j}} = \frac{k_{a,i} F_i C_{TOT} R_{max,i}}{R_{max,j}^2} R_j$$

We obtain:

$$\frac{\partial R_{PRED,TOT}}{\partial R_{max}} = \begin{bmatrix} \sum_{i=1}^{N} \frac{\partial R_i}{\partial R_{max,1}} \\ \vdots \\ \sum_{i=1}^{N} \frac{\partial R_i}{\partial R_{max,N}} \end{bmatrix} = \begin{bmatrix} \frac{\partial R_{PRED,TOT}}{\partial R_{max,1}} \\ \vdots \\ \frac{\partial R_{PRED,TOT}}{\partial R_{max,N}} \end{bmatrix}$$

For each time point of each sensorgram, we obtain:

$$\frac{\partial R_{PRED,TOT}}{\partial \boldsymbol{\theta}} = \begin{bmatrix} \frac{\partial R_{PRED,TOT}}{\partial k_{a1}} \\ \vdots \\ \frac{\partial R_{PRED,TOT}}{\partial k_{aN}} \\ \frac{\partial R_{PRED,TOT}}{\partial k_{d1}} \\ \vdots \\ \frac{\partial R_{PRED,TOT}}{\partial k_{dN}} \\ \frac{\partial R_{PRED,TOT}}{\partial R_{max,1}} \\ \vdots \\ \frac{\partial R_{PRED,TOT}}{\partial R_{max,N}} \end{bmatrix}$$

Which enables the computation of the gradient $\frac{\partial R_{TOT,pred}}{\partial \boldsymbol{\theta}}$. Computing this gradient at every time step of every sensorgram in the data set is necessary to compute the hessian matrix.

2. Supplementary Appendix B - Confidence intervals on the fractions

We define $\hat{F}_i$ as the estimated fraction of analyte i, i.e.:

$$\hat{\boldsymbol{\theta}} = \begin{bmatrix} \hat{F}_1 \\ \vdots \\ \hat{F}_N \end{bmatrix}$$

We propose an algorithm similar to the bisection method. Pose:

$$G(F_{i0}) = J(\theta)|_{F_i=F_{i0}} - J(\hat{\theta}) - F_{1-\alpha}(1, n-p) * \frac{n-p}{J(\hat{\theta})}$$

The boundary of the confidence interval is such that $G(F_{i0}) = 0$. If points a and b such that $G(a) > 0$ and $G(b) < 0$ are known, the algorithm consists in:

1. Compute $G\left(\frac{a+b}{2}\right)$.
2. If $G\left(\frac{a+b}{2}\right) < 0$, pose $b = \frac{a+b}{2}$. Otherwise, pose $a = \frac{a+b}{2}$.
3. Test for convergence, i.e. the algorithm can be stopped if $a - b < TOL$ or if $\frac{a+b}{2}$ is such that $F_{i0} > 1$ in the case of an upper bound or $F_{i0} < 0$ in the case of a lower bound.
4. If there is convergence, the boundary is given by $\hat{F}_i + \frac{a+b}{2}$ in the case of an upper bound or $\hat{F}_i - \frac{a+b}{2}$ in the case of a lower bound. Otherwise, return to step 1.
5. Repeat for every analyte and for upper and lower bounds.

To obtain starting points for a and b, we can:

1. Pose $a = 0.5\%$ and $b = 0\%$.
2. Compute $G(a)$.
3. If $G(a) < 0$, pose $b = a$, double a then return to step 2.
4. If $G(a) > 0$, the current a and b can be used as a starting point for the bisection algorithm.
